# Management of skin rash during egfr-targeted monoclonal antibody treatment for gastrointestinal malignancies: Canadian recommendations

**DOI:** 10.3747/co.v16i1.361

**Published:** 2009-01

**Authors:** B. Melosky, R. Burkes, D. Rayson, T. Alcindor, N. Shear, M. Lacouture

**Affiliations:** * BC Cancer Agency, Vancouver, BC; † Mount Sinai Hospital, Toronto, ON; ‡ QEII Health Sciences Centre, Halifax, NS; § McGill University Health Centre, Montreal General Hospital, Montreal, QC; || Sunnybrook Health Sciences Centre, Toronto, ON; # Robert H. Lurie Comprehensive Cancer Center, Chicago, IL, U.S.A

**Keywords:** Canadian recommendations, epidermal growth factor receptor inhibitor, egfri, anti-egfr, side effects, skin rash, dermatologic toxicity, proactive management, treatment algorithm, gastrointestinal malignancy, colorectal cancer

## Abstract

The epidermal growth factor receptor (egfr) is often overexpressed or dysregulated in a variety of solid tumours, including gastrointestinal (gi) malignancies. Agents targeting the egfr-mediated signalling pathway are increasingly part of the therapeutic armamentarium for the treatment of advanced lung, head-and-neck, and colorectal carcinoma. The egfr inhibitors (egfris) approved in Canada include the tyrosine kinase inhibitors erlotinib and gefitinib (in selected cases), and the monoclonal antibodies (mAbs) panitumumab and cetuximab. Although egfris have been proven effective in the treatment of a variety of malignancies, the entire class of agents is associated with a high prevalence of dermatologic side effects, most commonly skin rash. This reversible condition requires intervention in approximately one third of patients. A proactive, multidisciplinary approach to management can help to improve skin rash and optimize clinical outcomes by preventing egfri dose reduction or discontinuation. In addition, effective management and patient education may help to alleviate the significant social and emotional anxiety related to this manageable side effect, thus resulting in improved quality of life. The present article focuses on egfr-targeted mAbs for the treatment of gi malignancy, addressing the pathophysiology, clinical presentation, and incidence of skin rash caused by this class of agents. Recommendations aimed at establishing a framework for consistent, proactive management of skin rash in the Canadian setting are presented.

## 1. INTRODUCTION

The epidermal growth factor receptor (egfr) is a trans-membrane glycoprotein that is expressed in many normal human cells of epithelial origin, playing an important role in cell growth, differentiation, and proliferation. The receptor consists of an extracellular ligand-binding domain, a transmembrane region, and an intracellular tyrosine kinase domain 1.

In many solid tumours, including most gastrointestinal (gi) malignancies, egfr is overexpressed 2. Dysregulated egfr may result in uncontrolled cell growth, proliferation, and angiogenesis, and is associated with a poorer prognosis, manifested by increased metastatic potential and poorer overall survival (os) times 3. Thus, egfr is an ideal target for antitumour therapy. Inhibitors of this target can be broadly classified as either tyrosine kinase inhibitors (tkis) or monoclonal antibodies (mAbs), both of which can produce significant skin toxicity.

The egfr-blocking tkis have been the subject of several publications on skin rash, and therefore the present article focuses on management of rash resulting from egfr-targeted mAbs for the treatment of gi malignancy. Although the management of rash caused by tkis and mAbs is clinically similar, there are differences between these two classes of egfr-targeted agents with regard to the incidence, severity, and onset of this skin toxicity.

## 2. EGFR-TARGETED MONOCLONAL ANTIBODIES

The egfr-targeted mAbs are given intravenously and act by binding the extracellular domain, thus blocking ligand binding and tyrosine phosphorylation. In Canada, two mAbs have been approved for the treatment of metastatic colorectal cancer (mcrc): cetuximab and panitumumab (TABLE I).

Cetuximab (Erbitux: Bristol–Myers Squibb, Princeton, NJ, U.S.A.) is a recombinant human/mouse (chimeric) immunoglobulin G1 mAb that is administered once weekly. Cetuximab in combination with irinotecan (Camptosar: Pfizer Canada, Kirkland, QC) is indicated in Canada for the treatment of egfr-expressing mcrc in patients who are refractory to other irinotecan-based chemotherapy regimens. It is also indicated as monotherapy for egfr-expressing mcrc in patients with intolerance to irinotecan-based chemotherapy 4.

Panitumumab (Vectibix: Amgen Canada, Mississauga, ON) is a fully-human immunoglobulin G2 mAb administered once every 2 weeks. Panitumumab is indicated as monotherapy for patients with egfr-expressing mcrc and with non-mutated [wild-type (wt)] *KRAS* after disease progression on fluoropyrimidine-, oxaliplatin-, and irinotecan-containing chemotherapy regimens 5. Panitumumab became available in Canada in summer 2008 and was added to the Ontario provincial drug program in November 2008.

### 2.1 Efficacy of EGFR-Targeted mAbs for Third-Line Therapy of Advanced Colorectal Malignancy

The egfr-targeted mAbs have demonstrated efficacy in the treatment of advanced colorectal malignancy in a number of clinical trials (TABLE II).

In a randomized phase iii study of chemotherapy-refractory mcrc, panitumumab monotherapy almost halved the risk of disease progression as compared with progression in a best supportive care (bsc) control group [hazard ratio (hr): 0.54; 95% confidence interval (ci): 0.44 to 0.66; *p* < 0.0001]. No significant difference was observed in os, likely because of the high percentage (76%) of bsc patients who crossed over to panitumumab at disease progression. Objective response was observed in 10% of patients randomized to panitumumab and in 11% of patients crossing over to this agent, as compared with 0% in the bsc group 9.

A retrospective analysis of the phase iii study examined the influence of *KRAS* mutation status on the therapeutic efficacy of panitumumab. That analysis reported that progression-free survival (pfs) was significantly greater in patients with wt *KRAS* than in patients with a mutant gene (*p* < 0.0001). Median pfs in wt *KRAS* patients was 12.3 weeks for the panitumumab group as compared with 7.3 weeks for the bsc group. To account for potential tumour-ascertainment bias in favour of the bsc arm, an interval-censored sensitivity analysis was performed in which radiologic event times were moved to the closest assessment time pre-specified in the study protocol. That analysis showed median pfs times of 16 weeks and 8 weeks with panitumumab and bsc respectively (hr: 0.44; 95% ci: 0.30 to 0.63). No significant difference in os was observed between treatment arms for all patients (hr: 0.97; 95% ci: 0.79 to 1.18) or between *KRAS* groups (mutant gene—hr: 1.02; 95% ci: 0.75 to 1.39; wt gene—hr: 0.99; 95% ci: 0.75 to 1.29) 7. This report confirmed that, in terms of pfs, the efficacy of panitumumab monotherapy for mcrc is limited to patients with wt *KRAS* tumours. Accordingly, *KRAS* mutation status must be evaluated to optimize selection of patients with mcrc for panitumumab monotherapy.

The efficacy of cetuximab monotherapy was evaluated in a phase iii National Cancer Institute of Canada (ncic) trial that randomized chemotherapy-refractory mcrc patients to cetuximab monotherapy or bsc. Compared with bsc alone, cetuximab treatment was associated with significant improvements in pfs (hr: 0.68; 95% ci: 0.57 to 0.80; *p* < 0.001) and os (hr: 0.77; 95% ci: 0.64 to 0.92; *p* < 0.005). Median os in the cetuximab group was 6.1 months as compared with 4.6 months in the bsc group 10. This ncic trial did not allow crossover to active therapy for patients initially randomized to receive bsc alone.

A Cox model analysis of the study examined the predictive effect of *KRAS* mutation status on os and pfs. The authors reported that the effect of cetuximab was significantly greater in the wt *KRAS* group than in the mutant gene group both for pfs (*p* < 0.0001) and for os (*p* < 0.01). No significant difference in os as a function of *KRAS* status (wt vs. mutant) was observed in the bsc arm (hr: 1.01; 95% ci: 0.74 to 1.37; *p* = 0.97). The authors concluded that *KRAS* mutation status is a strong predictive biomarker and that mutation analysis can be considered a new standard of care in the selection of patients for egfr-targeted therapy 8. For this retrospective analysis, *KRAS* status was available for 69% of patients in the phase iii cetuximab monotherapy study as compared with 92% of the patients in the phase iii panitumumab monotherapy analysis discussed earlier 7,8.

Cetuximab plus irinotecan was studied in a randomized phase ii trial of third-line therapy for patients with mcrc: combination therapy was compared with cetuximab alone. Median time to progression was significantly greater in the combination arm (4.1 months) than in the monotherapy arm (1.5 months). Tumour response rates (22.9% vs. 10.8%) and median survival times were also significantly higher in the combination arm than in the monotherapy arm 6.

### 2.2 Adverse Effects of EGFR Inhibition

The toxicity profile of egfr-targeted mAbs excludes many of the severe side effects commonly observed with cytotoxic chemotherapy. As a class, however, egfr inhibitors (egfris) are characterized by cutaneous adverse effects, most commonly a papulopustular reaction involving skin 11. Skin rash is mostly mild-to-moderate in severity and requires therapeutic intervention in about one third of patients 12. Although the skin rash is self-limiting and usually resolves without scarring upon discontinuation of anti-egfr therapy 13, the condition can negatively affect treatment compliance and quality of life. In addition to leaving skin vulnerable to bacterial overgrowth and serious infection, skin rash can lead to dose modification or treatment discontinuation, thus potentially affecting the overall clinical benefits of this form of therapy.

In a post-approval survey, 76% of respondents reported holding egfris at some point during therapy because of skin rash, and up to 32% of physicians reported discontinuing egfri treatment altogether 14. Moreover, significant pain and pruritus, and anxiety related to the cosmetic appearance of the rash can negatively affect patient quality of life. Patients report that stinging or burning, irritation, dry eyes, pain, and sleep disturbances are the most significant symptoms 15. Proactive strategies for the management of egfri-mediated skin rash may help to maximize benefit for patients by minimizing the negative effects on quality of life and maintaining an optimal mAb dose.

The next section of this article reviews the pathophysiology, clinical presentation, and incidence of skin rash resulting from mAb therapy in colorectal cancer and presents a practical treatment algorithm summarizing recommendations—from a Canadian perspective—for the management of this skin toxicity. Also addressed are the importance of proactive management, the correlation between rash and efficacy of therapy, and the direction of future research in this area.

## 3. PATHOPHYSIOLOGY, CLINICAL PRESENTATION, AND INCIDENCE OF SKIN RASH

### 3.1 Pathophysiology of mAb-Mediated Skin Rash

**FIGURE 1:**
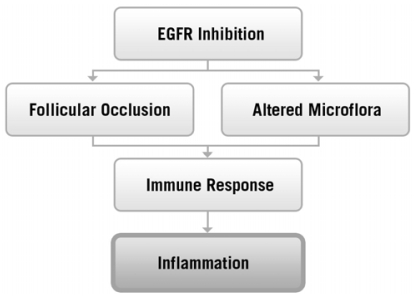
Pathophysiology of skin rash mediated by epidermal growth factor receptor (egfr) inhibition. (Adapted from Busam et al., 2001^19^.)

Epidermal growth factor receptor is normally expressed in the epidermis, sebaceous glands, and hair follicular epithelium 16, where it plays a number of important roles in the maintenance of normal skin health, including control of differentiation, protection against damage induced by ultraviolet radiation, inhibition of inflammation, and acceleration of wound healing 17. Although the exact mechanism of skin rash mediated by egfr-targeted mAb is incompletely understood, inhibition of egfr is believed to cause follicular occlusion and rupture because of premature epithelial differentiation and an increase in the expression of genes that stimulate inflammation, apoptosis, and cell attachment 12,18 (FIGURE 1). This altered permeability barrier may also allow for the promotion of bacterial overgrowth 20, further exacerbating cutaneous injury and development of the characteristic skin rash.

### 3.2 Description and Incidence of Skin Rash Mediated by EGFR-Targeted mAb

Treatment with egfri is associated with a spectrum of epidermal-derived toxicities. Although papulopustular skin rash is the most common skin toxicity associated with egfr-targeted mAbs (and the focus of this article), other less frequent side effects can include dry skin, pruritus, fissures, palmar–plantar rash, hyperkeratosis, telangiectasia, hyperpigmentation, blisters, mucositis, and pyogenic granuloma. Changes may also occur to the hair (for example, alopecia of the scalp or trichomegaly of the eyelashes) and nails (usually periungual manifestations such as paronychia) 17,21.

In general, skin rash associated with the use of egfr-targeted mAbs tends to be more severe and to occur with higher incidence than is observed with tkis. It also presents as a more purulent and pustular reaction which may require more aggressive interventions 13. Skin rash has been reported in 80%–90% of patients with mcrc treated with egfr-targeted mAbs as third-line therapy, with most cases being mild-to-moderate in severity (TABLE III). The rash occurs more frequently in areas of the face, neck, shoulders, upper trunk, and scalp 17 and other sun-exposed areas of the body 23.

### 3.3 Skin Rash Timeline

**FIGURE 2:**
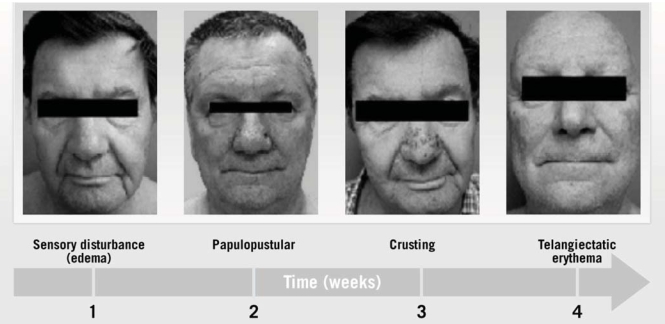
Characteristic phases of skin rash mediated by epidermal growth factor receptor (egfr) inhibition. (From Lacouture et al., 2007 24.)

An egfri-mediated rash generally follows a well-characterized clinical course (FIGURE 2). Within the first week of treatment, patients experience sensory disturbance with erythema and edema. From weeks 1 to 3, the papulopustular eruption manifests, followed by crusting at week 4 25,26. Despite successful treatment, erythema and dry skin may persist in the areas previously affected by the skin rash through weeks 4–6 27.

### 3.4 Manifestation of mAb-Induced Skin Rash

Based on phase iii study experience, cetuximab-induced skin rash appears to be dose-related 28 and generally evolves within 1–3 weeks of the start of treatment for mcrc 6. Panitumumab-associated rash also develops within the first 3 weeks of treatment for mcrc, with a median time of 14 days after start of therapy and a median time to resolution of 84 days after the last dose 9. Most patients with egfri-mediated skin rash exhibit some degree of spontaneous partial improvement during therapy, and the rash generally resolves completely and without scarring following cessation of the egfr-targeted drug 13.

It is important to note that no association has been observed between mAb-mediated skin rash and past or pre-existing skin abnormalities such as acne or ro-sacea 17,24. Additionally, terms such as “acne-like” or “acneiform” to describe this unique rash are incorrect and should be avoided, because histopathology does not support this association 19,29.

### 3.5 Grading EGFRI-Mediated Skin Rash

Accurate grading of egfri-associated skin rash should assist in optimizing treatment by allowing clinicians to determine the most appropriate interventional strategy for each patient. Data on the grading or severity of egfri-mediated skin rash have been collected primarily in clinical trials using the U.S. National Cancer Institute’s *Common Toxicity Criteria* (nci-ctc) version 2.0 22 or its *Common Terminology Criteria for Adverse Events* (nci-ctcae) version 3.0 30; however, this system was designed as a broad surveillance tool to monitor adverse events, and its usefulness for documenting skin toxicities related to egfris is questionable 31. For example, the nci-ctcae relies heavily on body surface area as a determinant of rash severity. This approach fails to account for the location of egfri-associated rash—generally confined to the face and upper trunk—and also does not consider subjective patient tolerability and discomfort.

**FIGURE 3:**
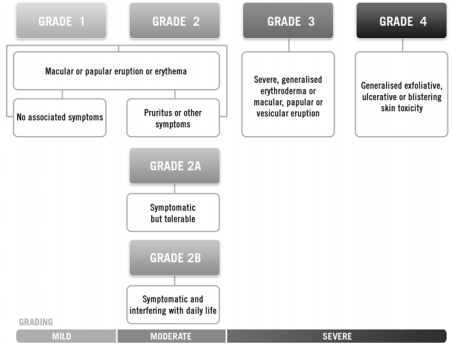
Modified grading system for skin rash mediated by epidermal growth factor receptor (egfr) inhibition. (Adapted from Pérez–Soler et al., 2005 29.)

An egfri-specific grading system, such as that proposed by Pérez–Soler 29 (FIGURE 3), more accurately reflects the specific nature of an egfri-mediated skin rash, thus allowing clinicians to individually optimize treatment. As well, such a grading system may provide a framework by which various therapeutic interventions may be compared, allowing optimal management strategies to be identified.

## 4. REVIEW OF EXISTING RECOMMENDATIONS ON THE MANAGEMENT OF EGFRI-MEDIATED SKIN RASH

### 4.1 Key Consensus Documents and Algorithms

Currently, the treatment of egfri-mediated skin rash is based principally on qualitative evidence and anecdotal experience that have yet to be validated by well-controlled randomized clinical trials. Yet, despite the absence of level 1 evidence, a number of consensus statements and algorithms 26,31–33 have been published to share best practices with the common goal of minimizing skin toxicities, improving quality of life, and maximizing egfri treatment benefits by minimizing the need for significant dose reductions (TABLE IV).

### 4.2 Proactive Management

In the clinical setting, up to 32% of physicians have reported discontinuing, and 76% have reported holding, egfri treatment because of skin toxicity 14. Interest in the development of primary pre-emptive strategies has grown as recognition of the spectrum of skin toxicity has evolved and so as to minimize dose reduction or treatment discontinuation, which both potentially compromise the clinical benefit of egfris. As a result, a small number of randomized controlled trials have been designed to evaluate primary preemptive strategies (TABLE V).

A controlled study called stepp (Skin Toxicity Evaluation Protocol with Panitumumab) is the first prospective trial designed specifically to compare primary pre-emption with reactive treatment for egfri-mediated skin toxicity 36. Patients receiving second-line folfiri (fluorouracil, leucovorin, irinotecan)–based chemotherapy plus every-second-week panitumumab (*n* = 32) or irinotecan-based chemotherapy plus panitumumab every 3 weeks (*n* = 26) were randomized to primary pre-emptive or reactive skin treatment. Patients randomized to the pre-emptive treatment arm received daily skin treatment from 24 hours before their first dose of panitumumab through week 6. Patients in the reactive treatment arm received treatment after development of skin toxicity. Skin toxicity treatment in the pre-emptive arm included skin moisturizer, sun-screen, 1% hydrocortisone cream, and doxycycline 100 mg twice daily. Treatment and timing of therapy initiation in the reactive arm were left to the discretion of the investigator. Recently presented results indicated that, as compared with reactive treatment, pre-emptive treatment reduced the incidence of grade 2 or greater skin toxicities by more than 50% without additional side effects. In addition, time to severe skin toxicity was significantly delayed in the pre-emptive treatment arm. The time to first occurrence of any grade 2 or greater skin toxicity was also significantly delayed in the preemptive arm.

Two randomized double-blind trials have examined the effects of prophylactic skin rash treatment. An 8-week trial studied prophylactic oral minocycline as compared with placebo for patients with mcrc preparing to initiate cetuximab therapy. Patients were also randomized to receive topical tazarotene application to the left or right side of the face. At weeks 1–4 of mAb treatment, the minocycline group (*n* = 24) had a significantly lower total facial lesion count and a significantly reduced incidence of moderate-to-severe itch as compared with the placebo group (*n* = 24, *p* = 0.05). Study authors also concluded that topical tazarotene was not recommended for the management of cetuximab-mediated rash because it provided no clinical benefit and was associated with significant skin irritation 34. In another double-blind trial, patients starting egfri therapy were randomized to tetracycline (500 mg twice daily) or to placebo for 4 weeks (*n* = 61). Although tetracycline did not prevent egfri-induced rash, a reduction in rash severity was observed. At week 4, grade 2 rash was reported in 17% of the tetracycline group and in 55% of the placebo group (*p* = 0.04). Treatment also improved certain skindex-16 quality-of-life measures, including skin burning or stinging and skin irritation 35.

Additional well-controlled prospective clinical trials are needed to further examine the potential benefits of primary pre-emption of egfri-mediated skin toxicities, and to establish a framework for consistent evidence-based treatment approaches based on biologic mechanisms. In addition, further evaluation of patient, nursing, and physician education tools is important and may aid in the promotion of prophylactic intervention, early recognition, and best practices in skin toxicity management, thereby maximizing potential clinical benefit from the egfri class of agents, reducing the risk of serious infection, and improving patient quality of life.

### 4.3 Rash-to-Survival Correlation

Available retrospective evidence suggests that the appearance and severity of skin rash is positively correlated with objective tumour response to egfri therapy and with os in mcrc. Data suggest that skin rash may serve as a surrogate marker of egfr-targeted mAb efficacy. In several analyses, both the presence and intensity of skin toxicity predicted objective tumour response in patients treated with cetuximab or panitumumab in mcrc. In patients receiving cetuximab monotherapy for refractory mcrc, longer survival times were observed in patients with rash of any grade as compared with patients experiencing no rash (*p* = 0.02) 37. In the pivotal phase ii trial of cetuximab in combination with irinotecan as compared with cetuximab alone in patients with mcrc, response rates in patients with skin reaction were higher than rates in patients without skin reaction (25.8% vs. 6.3% in the combination group, 13.0% vs. 0% in the monotherapy group; *p* = 0.005) 6.

**FIGURE 4:**
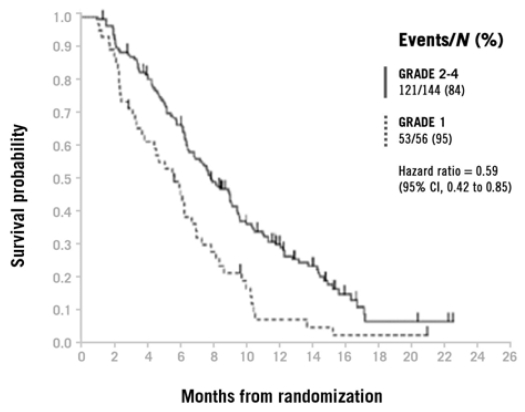
Overall survival by severity of skin rash in patients with third-line metastatic colorectal cancer receiving panitumumab. (From Van Cutsem et al., 2007 9.)

Similar results have been observed in phase ii and phase iii studies of panitumumab. An exploratory analysis of the pivotal phase iii trial of panitumumab for third-line therapy of mcrc observed a positive correlation between increasing rash severity and improved os (FIGURE 4) 9. In a phase ii study of panitumumab in mcrc, patients with grades 2–4 maximum skin toxicity had improved pfs (hr: 0.67; 95% ci: 0.50 to 0.90) and os (hr: 0.72; 95% ci: 0.54 to 0.97) as compared with patients experiencing grades 0–1 skin toxicity 38. These observations were confirmed in a phase iii open-label study in which improved pfs was observed for patients with grades 2–4 as compared with grade 1 skin toxicity 39.

Overall, these observations support the consensus that patients with mcrc receiving egfris who develop skin rash should be treated for the rash while continuing egfr-targeted mAbs, because these patients may derive the greatest clinical benefit from therapy. Note that these observations have yet to be validated by prospective trials, and it should not be assumed that egfr-targeted mAbs are ineffective in patients who do not develop rash.

### 4.4 Dose-to-Rash Studies

Retrospectively observed rash-to-survival correlations suggest that individualized dose titration based on the appearance and severity of skin rash may allow for optimization of egfri therapy and have led to the initiation of “dose-to-rash” trials (increasing the dosage until rash is observed) that are examining dose escalation protocols. The phase i/ii dose escalation trial in patients with mcrc with no or slight skin reactions on standard-dose cetuximab treatment, also known as the everest trial, supports the rash-to-survival correlation, with prospective data demonstrating an increase in rash severity and response rate with escalating dose of cetuximab^40^. Patients with mcrc with no or slight skin reactions after 22 days of standard cetuximab therapy were randomized to continue receiving a standard dose or to begin a dose escalation protocol that involved increasing the dose every 2 weeks until a grade 2 skin toxicity or a cetuximab dose of 500 mg/m^2^ was achieved. As compared with the standard-dose arm, the dose-escalation arm demonstrated an improvement in response rate (risk ratio: 30% vs. 13%). This finding supports the relationship between tumour response rate, egfri dose, and skin toxicity, thereby substantiating the correlation between egfri activity at the level of the egfr receptor on the skin and in the tumour.

Although the mechanism underlying the correlation between skin toxicity and tumour response is currently unclear, investigators have hypothesized that the rash may be a surrogate marker for degree of receptor saturation by the egfri agent. Accordingly, researchers have speculated that skin toxicity may aid in predicting which patients may preferentially or maximally benefit from egfri therapies. In addition, targeting doses to achieve a desired level of cutaneous toxicity may further increase the efficacy of egfris. Ongoing and planned dose-to-rash studies will further evaluate the potential benefit of dose escalation for patients with mcrc and no or mild skin toxicity from egfr-targeted mAbs. If these studies confirm a relationship between egfri dose and response rate, definitive studies using pfs as an endpoint will be justified 12.

## 5. CANADIAN RECOMMENDATIONS FOR MANAGEMENT OF EGFRI-MEDIATED SKIN RASH

Given that anti-egfr mAbs have demonstrated survival advantages in second- and third-line therapy of mcrc, the use of these agents continues to increase. Forthcoming randomized clinical trial results will also determine whether these agents are efficacious in the first-line setting. As the use of these agents expands in Canada, a better understanding of the associated skin toxicities and optimal patient management strategies is becoming increasingly important and remains a significant area of new educational need for many clinical oncologists, oncology nurses, and general practitioners.

### 5.1 General Pre-emption and Treatment Principles

The treatment strategies that follow are recommended, based on consensus, for pre-emption and management of mAb-mediated skin rash and other dermatologic toxicities associated with these agents.

- Patients should be advised to take appropriate sun protective measures, because sun-exposure can exacerbate rash severity on unprotected areas of the body 23.
- Patients should also be counselled to avoid activities and skin care products that dry the skin. Examples include long, hot showers; alcohol-based or perfumed products; and over-the-counter acne medication. Greasy ointments should also be avoided in favour of frequent moisturizing with alcohol-free emollient creams.
- For symptomatic relief, oatmeal baths can be soothing and are likely anti-inflammatory 41.
- Creams are more effective than lotions, and when kept cool (for example, refrigerated), they can provide symptomatic benefit.

### 5.2 Skin Rash Treatment Recommendations

**FIGURE 5:**
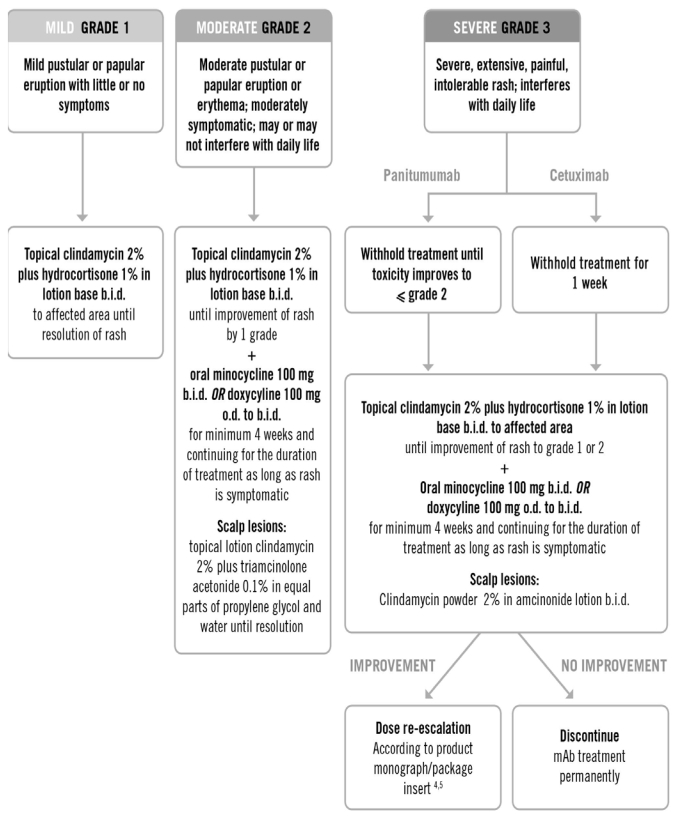
Treatment recommendations for rash mediated by monoclonal antibody (mAb) targeting of epidermal growth factor receptor, by severity. (Adapted from the BC Cancer Agency’s egfr inhibitors rash protocol.) o.d. = once daily; b.i.d. = twice daily.

Specific Canadian treatment recommendations are presented below according to grade of skin toxicity (FIGURE 5). Grades 1, 2, and 3 reflect skin toxicity that is mild, moderate, and severe respectively (adapted from nci-ctc). Although this grading system is primarily intended for skin rash on the face and scalp, the same grading can be applied to truncal rash, dry skin, pruritus, and nail or periungual changes.

- Topical 2% clindamycin is recommended for mild-to-severe skin rash, because this antibiotic has demonstrated favourable results, including a drying effect, on inflammatory pustules mediated by egfri therapy 42.
- The use of a low- to medium-potency topical steroid such as 1% hydrocortisone in a lotion base further enhances treatment of mild-to-severe rash by inhibiting inflammation 25.
- For moderate-to-severe skin toxicity, the oral semi-synthetic tetracycline antibiotics minocycline or doxycycline are recommended in addition to the topical treatments already described. Although egfri-mediated skin toxicity does not seem to involve infectious agents, the anti-inflammatory properties of the tetracyclines, via matrix metalloproteinase inhibition, may explain their effectiveness 43,44.
- Overall, management of skin rash should be individualized for each patient, depending on the type, severity, and location of the skin toxicity caused by anti-egfr therapy.
- Importantly, several situations warrant patient referral to a dermatologist. Clinicians may wish to refer if the skin toxicity does not improve within 1–2 weeks of treatment. Referral is also recommended if the patient is severely symptomatic (for example, if necrosis, blistering, or petechial or purpuric lesions are present) or if multiple hair, nail, and skin issues emerge 29. In general, if the skin toxicity has an uncharacteristic appearance or distribution (it just doesn’t look “right” or familiar), it is advisable to refer the patient to a dermatologist.

## 6. SUMMARY

Skin rash is a predictable but manageable side effect of anti-egfr therapy, including therapy with the egfr-targeted mAbs cetuximab and panitumumab. A proactive, multidisciplinary approach to pre-emption and therapy of skin toxicities will help to limit the incidence of severe symptoms, improve patient tolerance of therapy, maximize quality of life, and optimize clinical benefits of egfr-targeted mAbs by minimizing the need for dose reduction or early treatment discontinuation. The Canadian treatment recommendations and algorithm presented in this article represent the current consensus-derived best practice for treatment of skin rash mediated by egfr-targeted mAbs in patients with mcrc, and they are likely generalizable to other diseases being treated with these agents. Although well-designed prospective trials remain necessary to confirm best practices, educational tools such as this algorithm may serve to guide health care professionals and to inform patients with the goal of maximizing the benefits of egfri therapy. Establishing evidence-based approaches to the treatment of skin toxicities will become an even greater priority as the indications for these agents expand into earlier treatment and adjuvant settings for mcrc and other tumour types.

## Figures and Tables

**TABLE I:** Health Canada–approved monoclonal antibodies targeting inhibitors of epidermal growth factor receptor

Agent	Type	Indication	Dosing Schedule
Cetuximab	Mouse/human chimeric IgG1 monoclonal antibody	Third-line metastatic colorectal cancer, with or without irinotecan	Once weekly
Panitumumab	Fully human IgG2 monoclonal antibody	Third-line metastatic colorectal cancer monotherapy	Every second week

IgG = immunoglobulin G.

**TABLE II:** Pivotal phase iii and ii trial results of monoclonal antibodies targeting inhibitors of epidermal growth factor receptor in patients with third-line metastatic colorectal cancer

Reference	Regimens	Progression-free survival (pfs) or time to progression (ttp)	Overall survival	Response rate (rr)
Cunningham *et al.*, 2004 6	Phase ii cetuximab plus irinotecan vs. cetuximab monotherapy, all patients	Median ttp: 4.1 months (combination) 1.5 months (monotherapy) *p<*0.001	8.6 months (combination) 6.9 months (monotherapy) *p<*0.01	Partial rr: 22.9% (combination) 10.8% (monotherapy) *p<*0.007
Amado *et al.*, 2008 7	Phase iii panitumumab monotherapy vs. best supportive care (bsc) alone in patients with wild-type *KRAS*	Median pfs: 12.3 weeks (panitumumab); 7.3 weeks (bsc) *p<*0.0001	No significant difference (confounded by crossover design) *p<*0.0001	Partial rr: 17% (panitumumab), 22% in crossover group; 0% (bsc), 12% in crossover group
Karapetis *et al.*, 2008 8	Phase iii cetuximab monotherapy vs. bsc alone in patients with wild-type *KRAS*	Median pfs: 3.8 months (cetuximab); 1.9 months (bsc) *p<*0.0001	9.5 months (cetuximab); 4.8 months (bsc) *p<*0.0001	Overall rr: 12.8% (cetuximab); 0% (bsc) *p<*0.001

**TABLE III:** Incidence of skin rash in pivotal phase iii and ii trials for monoclonal antibodies targeting inhibitors of epidermal growth factor receptor in third-line metastatic colorectal cancer

Reference	Agent	Regimen	Patients with skin rash (%)	
			Any grade^a^	Grade 3 or 4^a^
Cunningham *et al.*, 2004 6	Cetuximab	With irinotecan	80	9
Van Cutsem *et al.*, 2007 9	Panitumumab	Monotherapy	90	14
Jonker *et al.*, 2007 10	Cetuximab	Monotherapy	89	12

a Determined according to the U.S. National Cancer Institute’s Common Toxicity Criteria 22.

**TABLE IV:** Summary of selected consensus-derived publications for the management of skin rash mediated by inhibitors of epidermal growth factor receptor (egfri)

Reference	Description
Segaert *et al.*, 2005 32	Consensus-derived manuscript and treatment algorithm developed by dermatologists, gastroenterologists, and oncologists at a European consensus conference on egfri skin toxicity held in 2004 in Brussels, Belgium
Lacouture *et al.*, 2006 33	Treatment approach, algorithm, and subspecialty clinic (series) for the treatment of cutaneous and ocular egfri toxicities; developed by hematologists, oncologists, ophthalmologists, and dermatologists meeting in 2005 in Chicago, Illinois
Eaby *et al.*, 2008 26 and Lynch *et al.*, 2007 31	Two interdisciplinary consensus statements featuring a 3-tiered grading system for decision-making and stepwise intervention for skin toxicities; developed following a multidisciplinary forum attended by oncologists, nurses, pharmacists, and dermatologists in 2006 in Chicago, Illinois

**TABLE V:** Summary of randomized double-blind trials evaluating primary preventive treatment strategies for skin toxicity mediated by inhibitors of epidermal growth factor receptor (egfri)

Reference	Population	Treatment	Effect of preventive treatment
Scope *et al.*, 2007 34	Patients with metastatic colorectal cancer preparing to initiate cetuximab	8 Weeks prophylactic minocycline vs. placebo	Significantly fewer total facial lesion counts; significantly reduced moderate-to-severe itch; topical tazarotene associated with significant irritation
Jatoi *et al.*, 2008 35	Patients starting therapy with egfri	4 Weeks of prophylactic tetracycline vs. placebo	Significant reduction in rash severity; improved patient-reported skindex-16 measures of skin burning or stinging and skin irritation
Mitchell *et al.*, 2008 36	Patients with metastatic colorectal cancer preparing to initiate panitumumab	6 Weeks of pre-emptive treatment vs. reactive treatment with moisturizer, sunscreen, hydrocortisone cream, and doxycycline	Significantly reduced incidence of grade 2 or greater skin toxicities; significantly delayed time to severe skin toxicity; significantly improved event-free probability for grade 2 or greater skin toxicity
